# Veterinary Medical Association of New Jersey

**Published:** 1896-06

**Authors:** 


					﻿NEW YORK COUNTY VETERINARY MEDICAL ASSOCIATION.
The regular meeting of this Association was called to order at the Academy
of Medicine, on Tuesday evening, May 5th, by President Dr. Huidekoper, at
8.45 o’clock. On roll-call the following members responded: Drs. J. S.
Cattanach, J. S. Cattanach, Jr., Delaney, Ellis, Ferster, Giffen, Gill, Huide-
koper, Hanson, MacKellar, Meher, O’Shea, Robertson, Sherwood, and
Turner (15). The minutes of the previous meeting were read and approved.
The Board of Censors having no business to report, and there being no
papers, the time was given to discussion, which was opened by Dr. Robert-
son, who asked whether the Board of Health had a right to take possession
of and destroy animals that show a reaction to the use of mallein, without
any other outward signs of glanders ? A general discussion followed on that
point and on the symptoms of glanders in its various stages, and, finally,
the following resolution was offered : “ That the chair appoint a committee
of three to communicate with the Boards of Health of the city of New York
and the State of New York, and to investigate the value given by law to the
inoculation of glanders by mallein, and the extent of authority of the Boards
of Health in quarantining and condemning suspicious cases of glanders.”
The chair thereupon appointed the following committee : James L. Robert-
son (Chairman), H. D. Gill, and Robert W. Ellis (Recorder).
Dr. Gill gave a few reports on the use of antitoxin in tetanus, with nega-
tive results. Dr. Hanson reported a case in which the symptoms became
aggravated immediately after the injection of the antitoxin, which did not
abate until relieved by death, which soon followed. Board of Censors, Dr.
O’Shea, chairman, reported that our long-hoped-for Jury Bill failed to pass
the Senate. He then read a list of names of men registered in New York
County since October 1,1875, followed by a report on the investigation made
by the committee, on cards that were handed in at the last meeting, to the
effect that none of the men to whom their attention had been called were
registered, but that they had so f^r failed to get an interview with them.
Moved and seconded that the report be accepted; carried.
Next in the order of business was the reading by the Secretary of the
resignation of J. H. Ferster, V.S. The chair then asked the meeting their
pleasure in the matter. Moved by Dr. Hanson and seconded by Dr. O’Shea
that the matter be laid over until the next meeting. Amendment by Dr.
Gill that it be acted upon at the present meeting. Vote on amendment lost.
Vote on the motion carried.
Moved and seconded that a committee of three be appointed by the chair
to draft resolutions for alteration of that section of the by-laws covering
the night of meeting; carried. The following committee was appointed :
H. D. Hanson (Chairman), J. S. Cattanach, and T. Delaney.
Moved and seconded that the Secretary be authorized to obtain a bill
from the Chairman of the Judiciary Committee for expenses of that com-
mittee ; carried. Dr. O’Shea then notified the chair that a committee from the
Horseshoers’ Association was outside, with the request that the V.M.A.N. Y.C.
indorse their candidate for master horseshoer on the Horseshoers’ Examining
Board, Mr. O’Neil, and that the V.M.A.N. Y.C. appoint one of its members
as candidate for Veterinary Examiner on said board.
Moved and seconded that the chair appoint a committee of three to
recommend the name of Mr. O’Neil to the Governor as a member of the
Horseshoers’ Examining Board, and with the power to appoint a veterinarian.
The following committee was appointed : Thomas Giffen (Chairman), H. D.
Hanson, and F. W. Turner.
Moved, and seconded that the members of the V.M.A.N.Y.C. elect a
veterinarian to the examining board at the present meeting; carried. Dr.
Gill was unanimously elected.
Moved and seconded that the meeting adjourn ; carried.
Robert W. Ellis, D.V.S.,
Secretary.
				

